# The lore of low methane livestock: co-producing technology and animals for reduced climate change impact

**DOI:** 10.1186/2195-7819-9-10

**Published:** 2013-10-17

**Authors:** Ann Bruce

**Affiliations:** ESRC Innogen Centre, Old Surgeons’ Hall, High School Yards, The University of Edinburgh, Edinburgh, EH1 1LZ UK

**Keywords:** Climate change, Sheep, Cattle, Methane, Genetics, Co-production

## Abstract

Methane emissions from sheep and cattle production have gained increasing profile in the context of climate change. Policy and scientific research communities have suggested a number of technological approaches to mitigate these emissions. This paper uses the concept of co-production as an analytical framework to understand farmers’ evaluation of a 'good animal’. It examines how technology and sheep and beef cattle are co-produced in the context of concerns about the climate change impact of methane. Drawing on 42 semi-structured interviews, this paper demonstrates that methane emissions are viewed as a natural and integral part of sheep and beef cattle by farmers, rather than as a pollutant. Sheep and beef cattle farmers in the UK are found to be an extremely heterogeneous group that need to be understood in their specific social, environmental and consumer contexts. Some are more amenable to appropriating methane reducing measures than others, but largely because animals are already co-constructed from the natural and the technical for reasons of increased production efficiency.

## Introduction

Sustainable intensification of agriculture (Foresight. The Future of Food and Farming 2011) has become one of the major policy and research themes in the UK. It aims to achieve increased food production while limiting impact on the wider environment and ecosystems. While the term 'sustainable intensification’ is contested (Garnett & Godfray 2012), the approach has focussed attention on the importance of agriculture, as a source of food, a cause of environmental damage and provider of ecosystem services. In the context of animal agriculture, animal welfare is also an important factor. This complexity of objectives – providing food, ecosystem services and landscapes, while reducing pressure on the environment, and mitigating and adapting to climate change – presents a challenge to farmers and provides the canvas for this examination of animal-technology co-productions.

Greenhouse gas emissions from agriculture, particularly methane emissions from sheep and cattle have gained an increasing profile in the policy and academic worlds since the publication of a seminal report from the Food and Agriculture Organization of the United Nations (FAO) on the environmental impact of farm animals (Steinfeld *et al.*^2006^). From a climate change point of view, methane and nitrous oxide are both important but this paper will focus only on methane. Methane from ruminant animals (e.g. sheep, cattle, goats, but not pigs) is a product from the digestion of grass, and is estimated to be around 25 times more powerful as a greenhouse gas than carbon dioxide (Industry Delivery Partners 2011). Put simply, in terms of the amount of greenhouse warming potential generated per tonne of meat produced, pig meat produces about 4 tonnes as compared to nearly 15 tonnes for beef and nearly 16 tonnes for sheep (Genesis Faraday Partnership 2008 based on analysis by Cranfield University). In the UK, a key climate change policy event was the passing of the UK Climate Change (2008) Act. This Act mandates a reduction in greenhouse gas emissions from the UK by 80% by 2050 (from 1990 levels). Interim targets for different sectors of the economy have been set by the UK Climate Change Committee, a body created by Government to advise on how this reduction can be achieved. Emissions attributed to the agricultural sector are estimated at around 8% of total UK greenhouse gas emissions, with 38% derived from methane (Committee on Climate Change 2010).

A range of responses have been suggested to achieve reductions in methane emissions from sheep and beef cattle. These include measures to increase the efficiency of production, for example by improvements to animal health, reproduction and genetics. Another approach is to manipulate methane emissions, for example by use of feed additives, using grasses which result in lower methane releases or by vaccinating the rumen to alter the bacterial composition. The agricultural industry has responded by developing a greenhouse gas action plan with targets and priorities (Industry Delivery Partners 2011). These include a wide range of actions, such as improved animal nutrition and health, the use of genetics and improved grassland management. The plan has been developed through a partnership, which includes a large number of agricultural organisations, such as the National Farmers Union, as well as more ecologically-focussed organisations, such as the Farming and Wildlife Advisory Group and Elm Farm Organic Research Centre.

(Moran *et al.*^2011^) have calculated Marginal Abatement Cost Curves that highlight actions expected to give the best greenhouse gas response with least damaging economic impacts (or even in some cases positive impacts). They identify feed additives and genetic selection of beef cattle as measures with apparently good potential. Life cycle analyses developed by Cranfield University (Genesis Faraday Partnership 2008) highlighted the impact of genetic change, calculating that using genetics has reduced greenhouse gas emissions from pigs by 15% between 1988 and 2007. Beef cattle and sheep methane emissions were reduced by less than 1% during the same period, attributed to the slower adoption of genetic evaluation in these sectors. Jones *et al.* (2013) combine expert and farmer opinions and identify using legumes in grass mixtures as ranking highly as a way of mitigating greenhouse gas emissions in UK sheep production. Gill *et al.* (2010) advocate expressing efficiency in terms of production of 'human edible food’, to acknowledge that ruminants convert a non-human edible food (grass) into a human edible food. Garnett has written extensively on the subject (e.g. Garnett 2011) highlighting the potential adverse environmental and ethical consequences that may arise from excessive focus on production efficiency as a way of achieving methane reductions. A number of authors have linked production to consumption patterns and advocated reduction in meat consumption in rich, Northern countries as a way of reducing greenhouse gas emissions (e.g. Garnett 2011).

While the entire food chain is important and is also influenced by consumption patterns, this paper will focus mainly on the farm end of the chain, touching on consumers and the wider food chain only in so far as they influence on-farm activity. The framework of co-production of the animal and the technical, will be used in this paper. A focus on co-production avoids the tendency of veering either too much into the realm of a technical fix, or of emphasising the social at the expense of the natural (Goodman 1999). Aspects relating to methane as a climate change gas will be foregrounded as the context for co-production. I will first locate this paper in the co-production and climate change literature and then examine how animal-technical co-productions narrativise the conceptualisation of methane and how methane is understood by farmers.

### Ideas of co-production

The concept of co-production in this paper is situated in animal geography (e.g. Holloway 2007). Co-production is also a concept used extensively in science policy studies (Jasanoff 2006) and innovation studies (e.g. Schot & Rip 1996), although with somewhat different emphases (Doubleday 2007). The science policy approach focuses on how categories of natural and social are brought into being; a process which involves the “intertwining of the cognitive, the material, the social and the normative” (Jasanoff 2006, p6). A different (but complementary) understanding of co-production comes from innovation theory. Here the co-production refers to the way in which technological change is brought about by merging technical innovation and social innovation–processes found to be almost an inevitable part of technological change (e.g. Schot and Rip 1996).This latter approach draws on the frequent finding that impacts from technological innovation do not just happen, but rather require the active involvement of a range of different stakeholders, including end-users, to bring them about. When considering sheep and beef cattle, co-production can be understood in both the above senses. Using the former approach, the emphasis is on different concepts of animal breeding and ways of evaluating animals. Using the latter approach, the focus is on social structures enabling the development of methods of estimating genetic value, the distribution of 'genetic material’ (either as animals or in the form of semen), and so on.

The animal geography approach to co-production has been influenced by the perspective that technological change in agriculture is bringing about changes in human-farm animal relationships in ways that result in reconstituting the animals (Holloway & Bear 2011). Holloway (2007) gives a good example of this reconstitution from studying robotic milking of dairy cows. While the robotic milking system is intended to allow cows the freedom to act naturally and be milked to the cow’s agenda, cows that do not adapt to the robotic milking system are culled. In this way the dairy cows are co-produced to fit the robotic milking system.

In comparison to farming of other species, sheep and beef cattle in the UK have been relatively little impacted on by technology. One of the main technologies applied is genetics. Holloway and colleagues have extensively examined the social construction of genetics and breeding value in the context of beef cattle. Holloway (2004) demonstrated how animals are evaluated by farmers in a show ring context, where aesthetics as well as utility become important. In contrast, at the crux of technological genetic evaluation are Estimated Breeding Values or EBVs, statistically determined quantifications of genetic value. However, the link between an EBV and the genetic composition of the animal is not necessarily taken for granted or accepted by farmers (Holloway 2004). Even within the context of EBVs, genetic evaluations are not a single entity. Holloway *et al*. (2009) describe separate evaluations for meat production and calf production (Beef Value and Calving Value indexes, respectively), as well as the way in which individual animals are identified as contributing particular genetic qualities to the herd. Holloway & Morris (2008) demonstrate that animals consist not only of the material (the animal) but also the genetic information that goes with them. Holloway *et al.*^2011^) examine how values attached to animals are produced within, and affect, knowledge practices and describe the entangling of 'traditional’ and 'genetic’ modes of valuation. Genetic variation can also be viewed more broadly as a symbol of the farm family’s success (Convery *et al.*^2004^).

As with Holloway’s (2004) pedigree breeders, Calvert (2013) emphasises the importance of the concept of 'breed’ and its social construction within cattle breeding practices in the USA. Grasseni (2007) perceptively observes the dichotomy between the need to create a 'breed’ from individual animals of similar appearance, and selecting outliers from this group to produce the next generation, emphasising specific characteristics. Uniformity is first needed, in order then to identify distinctiveness. Breed can also be used to subvert technical expertise. Grasseni (2007) describes how dairy cattle breeders, in Italian mountain regions, deliberately undertook experimental crossing with a range of different breeds as a way of emphasising their disengagement and independence from agricultural and breeding advisors.

. Once established, a breed identity must also be maintained. Holloway *et al.* (2009) examine the pivotal role of UK breed societies in delineating a breed, by controlling entry to the breed and recording relationships among individuals in the breed. They suggest that breed societies, by their actions, are also implicated in the co-production of both humans and farm animals. Calvert (2013) identifies similar practices involved in pedigree Aberdeen Angus breeding in a more industrialised context in the USA.

Beef cattle and (to a lesser extent) sheep genetic practices rely on more than local knowledge. Gibbs *et al.* (2009) note how specialist genetics knowledge can be located at a considerable distance from farms in corporate facilities, highlighting the digital and mobile nature of EBVs. Once the 'body’ of an animal has been converted into computerised 'data’, it can be readily shared widely across computer networks.

Grasseni (2007) emphasises the management changes that become necessary as a result of adopting expert-led breeding programmes, for example, the resulting increase in size of cows leading to the need for better diets and increased stall sizes, and the need for infrastructure to supply semen. Thus, one technical change becomes predicated on a whole range of new practices and infrastructural accommodations in a form of animal-technical co-productions. In her specific case study of dairy cows in Italy, the adoption of standards and technical approaches also led to the need for professional specialisation between family members, and increased liaison with a wide-range of organisations such as vets and advisors on breeding and nutrition. The introduction of technical genetics, therefore, did not exist in isolation but required a reconceptualisation of the whole farming practice.

Beyond the co-construction with genetics, multiple and complex relationships have been found between humans and farm animals, both within the context of farmer relationships with farm animals (Riley 2011, Wilkie 2005, Convery *et al.*^2004^) as well as the relationships with farm animals of a range of different publics (Macnaghten 2004). These emphasise the extension of relationships beyond a merely economic, production orientation.

Ruminants, as grass-eating animals, are often associated with particular environments. Ruminants can be considered important for maintaining biodiversity and identified with a sense of the local (Evans *et al*. 2003; Yarwood & Evans , Yarwood & Evans Yarwood & Evans 2003). Yarwood & Evans (2006) suggest co-production of animal and locality takes place at the breed level. Gray (1998) goes further and suggests that for hill sheep farmers, knowledge of local sheep becomes essential in the breeding of specific flocks for specific places, with sheep becoming adapted or 'hefted’ to specific areas of land within a region. This type of conceptualisation is not necessarily applicable across national boundaries, for example, according to Saltzman *et al.* (2011) cattle are perceived to belong to Swedish nature, but as foreign to Australian nature.

. Haggerty *et al*. (2009) stress the dynamic relationship among landscapes, animal bodies and climate in a New Zealand sheep farming context. These relationships in turn are sites of contestation of what constitutes 'good’ farming (Burton et al. 2008) and have influenced the development and shape of sheep farming. As a result, Haggerty *et al.* (2009) argue that New Zealand sheep farms have developed in two different directions. Positive re-enforcement between productivism and the accrual of economic and cultural capital led some sheep farmers in a productivist direction, where improving productivity implied improving the animal body. Yet others responded to environmental and animal health challenges that began to arise by moving to new market linkages, with alternative conceptions of what is the 'good’ farmer. This bifurcation is also seen elsewhere. Grasseni (2007), for example, argues that breeding technologies are used by marginal farmers either as a means of converting to intensive farming or to pursue their identity as champions of both genetic and cultural capital.

This emergence of alternative conceptions of the 'good’ farmer engenders different practices of co-production with more emphasis on the consumer. Stassart & Whatmore (2003) provide a detailed analysis of the development of new alignments between consumers and producers, in the context of Belgian beef production. Stressing the ontological continuity between human and animal bodies, and responding to anxieties around industrial meat production, they suggest that meat is a co-production of the social and the natural, where healthy food is predicated on healthy animals. However, this co-production requires the complicity of a number of intermediaries, including butchers and certification bodies.

Co-productions have been identified between animal and technical (e.g. Holloway 2007), animal and production systems (e.g. Haggerty *et al.*^2009^), animal and environment (e.g. Yarwood & Evans 2006) and animal and consumer (Stassart & Whatmore 2003). This paper seeks to extend this range of examinations to consider inter-relationships with technologies to reduce methane emissions. I will firstly examine the literature relating farmers to climate change and to pollution more generally.

### Farmers, pollution and climate change

Greenhouse gases can be considered a form of pollution. The seminal work of Douglas (1966) emphasised the moral nature of pollution. Pollution, she found, was culturally identified as something to be disapproved of; as 'matter out of place’. This moral aspect to pollution in a farming context was echoed by Lowe and colleagues (Lowe *et al.*^1997^) studying water pollution from dairy farming. They identified two possible ways in which pollution could be framed; either as an unavoidable side effect of valuable production activities or as a morally discreditable practice, something that attracts blame. They explain “When defending themselves against charges of damaging the environment we found that farmers were able to draw on a rich repertoire of justification, which essentially naturalized their actions” (p192).

From this same empirical work, Ward *et al.* (1998) reported how dairy farmers tended to modify their practices to reduce water pollution in response to regulatory pressures, but in a rather “begrudging” (p1172) way partly because farmers perceived criticism as expression of a wider 'anti-farmer’ attitude in society rather than valid comment on sources of pollution. Ward *et al.* suggested that farmers responded in an 'add-on’ and 'end-of-pipe’ way rather than seriously engaging with the sources of pollution. Similarly, Ward *et al*. (1995; 1207) reported that “the group felt that agricultural pollution was far less of a problem than industrial pollution and suspected that farmers were being more strictly regulated because they were 'easy targets’.

This refusal to accept the 'pollution’ framing for farming activities is not restricted to the dairy farms studied by Ward *et al.* (1998). Diffuse water pollution from agriculture, in particular, is often not visible and its impacts are also not visible. Therefore, farmers tend not to be convinced that there is a problem or that they are part of the problem and can do something about it (Blackstock *et al*. 2010).

Climate change can also be viewed in terms of a moral issue, but it is more often conceived of in terms of risk. Survey evidence suggests wider publics are concerned about climate change and perceive the effects of climate change to pose a potentially serious risk, but this risk is expected to impact on other people. The risks to individuals themselves are perceived as being low (Spence *et al.*^2010^, Spence & Pidgeon 2010). Furthermore, climate change may even be perceived as having beneficial effects locally, e.g. due to warmer weather (Spence & Pidgeon 2010). Little research evidence exists specifically on farmer approaches to climate change. However, in common with wider publics, Holloway & Ilbery (1996) found UK arable farmers identified both beneficial and harmful effects of global warming. Holloway (1999) noted how arable farmers stressed the long timescales involved and difficulty of planning for unpredictable local climatic changes, as well as a certain amount of scepticism regarding the existence of climate change. Barnes and Toma (2012) surveyed 540 dairy farmers in Scotland and found only 48% agreed that temperatures would rise in the future. Survey data from Californian farmers (Haden *et al.*^2012^) suggests that farmers align with wider publics in perceiving climate change impact as being something primarily affecting other people.

Kahneman & Tversky (1982) identified that people generally will take risks to avoid losses, but are more averse to risking what they already have in order to gain more. Spence & Pidgeon (2010) apply these findings to attitudes to climate change and suggest that since wider publics do not perceive climate change as a personal threat, they are less likely to wish to risk what they have at the moment, in order to take action to mitigate climate change. Haden *et al.* (2012) extend this approach to farmers, and identify that Californian farmers were more likely to undertake practices to adapt to climate change since this is seen as a personal threat, rather than adopt mitigation practices which rely on a wider sense of doing good for others. Therefore, Haden *et al.* (2012) suggest that farmers are more likely to adopt mitigation practices which also have a tangible benefit to the individuals involved (e.g. nitrogen management, using less energy) than those of more general impact.

Actions of farmers can also be influenced by consumer pressure. Consumers in the UK increasingly associate perceptions of food quality with perceptions of the natural (e.g. Murdoch *et al.*^2000^) linking into ideas about the benefits of local production, embedded in local geographies. Eden *et al.* (2008) established that farmers markets are valued, in part because they are perceived to reconnect the consumer and the producer. Features such as dirt on vegetables, was used as evidence of local, authentic production. Gilg *et al.* (2005) argue that a shift to sustainable consumption is likely to be a component of a wider shift to sustainable lifestyles, but this shift is predicated on feeling that actions taken will be effective and have a positive impact on the environment. However, Barr *et al.* (2011) found that climate change mitigation was unimportant in the context of sustainable consumption, noting that 'climate change is for all intents and purposes, a relatively minor issue when it comes to embedding environmental practice’ (p 3025). This evidence suggests that consumers will not readily identify food consumption with climate change mitigation activity. Most consumption, however, is the end result of a long and often complex food chain. Green & Foster (2005) therefore argue that sustainable food production requires consideration of more than production and needs to include socio-technological practices across the entire food production system.

The research reported here extends the research on water pollution, climate change and consumer links with producers to consider methane emissions from sheep and beef cattle. I examine how farmers conceptualised methane, its role in climate change and potential mitigation methods. The research contributes to the body of knowledge around co-production by examining concepts of animals in a novel context and in the face of a novel challenge.

### Research method

The research sought to elucidate how individuals understand their world and their context and a qualitative methodology was adopted. I conducted 42 semi-structured interviews with farmers and other actors in the farming sector between September 2010 and March 2011. To reduce potential bias, a semi-structured interviewing approach provided a basic structure to the interviews and allowed greater flexibility to deeper explore specific items. Questions were focussed around how breeding replacements were chosen, how markets for breeding animals and meat influenced the breeding decisions, the perceived importance of environmental issues, and more specifically, the perceived importance of methane emissions as a factor to be considered. Interview schedules were informed by four preliminary, open-ended, scoping interviews with a range of industry representatives, including a representative of the meat industry, a commercial advisor to farmers, a specialist in native breeds and a provider of social support to farmers.

The 42 respondents included 30 sheep and beef cattle farmers and 12 representatives from the wider industry (vets, consultants, meat processors, breeding advisors, researchers). In order to avoid interviewing only elite farmers, interviewees were identified through a range of different 'gate-keepers’ including snowballing from preliminary and main interviews, recruitment through veterinary practices and farmers living near existing contacts in rural areas. Interviewees were purposively sampled to provide diversity and avoid bias as far as possible (Ritchie *et al.*^2003^). To this end, particular efforts were made to ensure representation from organic and environmentally-oriented farmers. Farms were located in a diverse range of environments, including upland and lowland, moor and marsh. A specific strategy was to target several different geographical areas within England and Scotland as a way of achieving variation in types of farming enterprise.

Farmers were interviewed from a wide-range of production systems, sizes and producing for different markets. Markets included producing breeding animals for direct sale or sale by auction; sale of meat animals either to other farmers for growing-on ('store’ animals), or as animals for slaughter; through auction, direct to meat processors , for specific supermarkets, or meat for direct sale in farm shops, to hotels or by post.

On approach for interview, farmers were given an information sheet outlining the purpose of the interview, including its focus on methane. The interview situation was not treated as a data mining exercise but rather that meaning was to be created in the interview encounter (Holstein & Gubrium 2002).

Interviewees included specialist sheep and beef farmers, but many farmers kept both species. Four dairy herds were included as producers of beef from beef-cross-dairy animals. Farms ranged in size from 4–1,000 beef cows and 8 – 1,300 ewes. Farmers were invited to complete a questionnaire to provide further information on the interview sample. 23 farmers completed this questionnaire. From these, it transpired that 30% of the farms self-identified as hill farms, 52% as not hill farms, with the remainder considering themselves marginal or upland farms. The farms relied heavily on animals for their income with 70% indicating that more than 50% of their finances came from animals. The majority of farms (91%) employed up to 5 people and most (83%) reported frequently using computers. Only 9% reported rarely using computers, and in at least one case this was due to the unavailability of broadband in the area. 43% of the farmers were aged 35–54, with 17% aged over 65 and 9% less than 35. The farmers were very experienced, 87% reporting more than 10 years of farming experience and 57% more than 30 years. On the basis of interview information, around 50% of farmers made use of technical genetic evaluations in the form of Estimated Breeding Values for beef, but less than 20% for sheep. However, this statistic is difficult to interpret as the extent of reliance on the Estimated Breeding Values varied. Some farmers stated they were primarily interested in the appearance of the animal but would also look at the Estimated Breeding Value.

Interviews with farmers were all conducted by the author and primarily face-to-face on farm, although a small number were undertaken as telephone interviews for logistical reasons. Most other respondents were interviewed in their place of work or by telephone. Interviews were recorded (with permission), transcribed and then analysed inductively, seeking to identify themes across the interviews. The interview data are not presented as statistically valid samples, but rather as a rigorous engagement with as wide a range of views as possible. Quotes used in this paper are illustrative of themes identified in the transcripts. Interviewees are identified by number and farming-type. Preliminary findings were presented to a one-day stakeholder workshop attended by 10 stakeholders and 2 academics. Feedback from this workshop was taken into account in finalising the analysis.

This qualitative approach has enabled understanding of how farmers view technological approaches to mitigating methane in their specific situations and to drawn inferences on how animals are conceptualised in a range of different circumstances. The data collection method does not allow for in-depth study of co-production in one particular context but is powerful in drawing on a large range of different contexts for a meta-level analysis.

### Research findings

In analysing the data, I have endeavoured to link farmer knowledge about animals with technical knowledge about animals. In doing this, I have paid attention to the different ways of knowing and the different objectives of the farmers. Furthermore, grazing animals always exist in specific natural and consumer environments, which impact on how farm animals are conceptualised.

The policy imperative to reduce methane emissions has tended to focus on solutions that depend on improving efficiency of production in terms of the amount of methane produced per unit of meat produced. Efficiency measures include a range of practices such as use of genetics to increase growth rate and efficiency of feed use, and improved reproductive performance and health. Beyond these a range of specific measures to reduce methane have been suggested, notably different varieties of grass and feed additive to reduce methane emissions, and alteration of rumen bacterial populations by vaccination to reduce methane emissions. Some farmers embrace this emphasis on efficiency and respond by adopting a range of efficiency enhancing measures and innovations. However, the motivation for such actions is driven more by economic considerations rather than methane reduction.

Cattle and sheep were constructed by farmers as means of providing a livelihood from a particular physical location. These were often in harsh environments particularly where sheep were concerned. In these situations, famers tended to emphasise the coproduction of their animals with the environment, such that their animals were particularly suited to the environmental niche they found themselves in. Manipulation of the animals, and to a large extent, animal husbandry methods, was considered risky, and therefore resisted.

Animals were also co-constructed as meat, particularly by farmers who sold meat products direct to consumers. For these farmers, demands of consumers and consumer perceptions of good quality meat became critical in determining desirable characteristics of their animals. These desirable characteristics were often in opposition to measures required to reduce methane emissions.

In the next sections, I will explore in more depth these three types of production, namely: focus on production efficiency, focus on production in demanding environments and focus on production for consumers. Each of these contexts draws out different aspects of animal-technical co-productions. By selecting themes that cross a range of farming approaches, I draw some generic principles that are likely to apply more widely across farms. While individual farms may emphasise one particular characteristic (like productivity) my broad-scale approach allows farms to appear in multiple categories, reflecting the complex ways in which individual farms are addressing the challenges of producing food, while minimising ecological impact and reducing greenhouse gas emissions. Finally, I will consider how farmers make sense of methane, demonstrating how this is conceptualised as an integral and inevitable part of an animal that cannot be disaggregated from the animal, rather than as a pollutant, by-product of beef and sheep-meat production. The contrast between the policy imperative to reduce methane and the farmers constructions of their animals are then highlighted in a final section.

### Animals and efficiency of production

Climate change policy (and much of the associated natural science research) agenda is predicated around a description of the methane challenge as one that is technocratic in nature. Such a framing emphasises the ability of technology to reduce the amount of methane produced per animal and hence reduce climate change impact, in large part by increasing the efficiency of production. The implication is that the material animal can be disaggregated (cognitively) into its component parts and these parts manipulated to create a new animal that is more desirable from a climate change point of view.

For farmers with a predominantly productivist approach to agriculture, the application of technology was perceived as a normal part of efficient farming. Emphasis was placed on the animal as a production unit and prominence was given to terms used in accountancy, such as gross margins and efficiency of resource use, as keys to evaluation of a 'good’ farmer.

Individual breeds were highlighted, but cross-breeding was also practiced and hybrid or 'synthetic’ breeds specifically developed. Market competitiveness was emphasised as evidenced in the following quote: “*There will be a lot of breeders select purely on looks of an animal. Our breeding aim of our breed is to try and improve the profitability of our customers, which is completely different to any other pedigree breeder in the country*” (interview 102^a^, sheep breeder). Similarly EBVs were embraced, for example, “*I want to know how that animal is going to perform, what it’s going to give me on my farm, so EBVs just seemed a very natural way of doing that really*” (interview 99, beef farmer). The focus was on performance measures rather than appearance, and economic performance rather than aesthetics. Any technologies advocated to reduce methane that also improved efficiency of production were seen as acceptable. New grass varieties producing less methane and new feed additives to reduced methane production would be acceptable as long as economic efficiency was unaffected or improved. Use of vaccines to alter rumen microbial populations to reduce methane emissions could be accepted subject to impact on overall efficiency and financial impact. Genetic techniques and disease reducing measures were already incorporated into farming practices.

The animal was perceived as an efficient converter of grass to meat (although often with additional cereal diets provided) and the emphasis was on, for example, residual feed intake (how much feed is needed beyond that strictly necessary for growth). Other technologies to increase efficiency were adopted as appropriate, e.g. automatic oestrus detecting equipment in order to increase the number of cows achieving pregnancy quickly and enabling use of high genetic merit sires. Unlike dairy cattle, beef cattle are not frequently observed by humans, therefore oestrus detection tends to be left to a bull. However, using a bull rather than artificial insemination (AI) limits the ability to use sires evaluated as having high genetic merit.

One prominent trend was towards minimising human interventions and hence improving economic efficiency. As an example, one strand of sheep breeding focuses on selecting animals that are robust, disease resistant and requiring minimal human assistance (e.g. with lambing), and even shedding its own wool (the cost of shearing exceeding the value of the wool gathered at the time of interviewing). While the methods used in this approach are very much technocratic, emphasising selection on the basis of numerical recording; the target traits (lambing, diseases, wool shedding) emphasise a return to the natural. As with the use of robotic milking (Holloway 2007), technology is used to reduce the amount of human intervention. However, in the case of introducing wool-shedding, a more radical physical change is introduced to the animal body, although the wool-shedding characteristic is introduced through a wool-shedding gene that occurs naturally in some breeds of sheep. The sheep is, then, a co-production of the material, physical characteristics that enable this modification to the sheep body, cognitive processes that value the 'natural’, but with a social emphasis on economics of producing sheep for meat, the high cost of labour and normative evaluations that recognise the appropriateness of using technology to achieve these aims.

Although the climate change policy and research focus has been largely on beef produced from beef cattle, in the UK around 50% of beef is supplied from dairy herds, much of it as beef-cross-dairy calves used to produce the pregnancies that ensure milk production. In terms of formal methane accounting, emissions are often completely attributed to the dairy cow. Nevertheless, beef-cross calves will produce methane, and are therefore, also an appropriate target for methane reducing measures. Among the dairy farmers interviewed core activity was seen as producing milk, with the beef-cross calf a 'by-product’ or more positively 'icing-on-the-cake’ and calves sold on to others for rearing as quickly as possible. The emphasis was on the cow as a reproductive unit, and AI semen was selected on the basis of good fertility and ease of calving.

Beef-cross calves were conceptualised by dairy farmers largely by their impact on the dairy cow, rather than as themselves. This construction of the calf is very different to that of farmers with beef production as part of their core objectives. For dairy farmers there is a clear distinction between 'valuable’ (dairy) calves and 'disposable’ (beef-cross and dairy bull calves) produced largely due to the specialisation inherent in current dairy farming practices. Manipulating the efficiency of the beef calf by selecting sires with genetic potential for fast, efficient growth was of little or no concern. In this way the calf is co-produced specifically within a type of production system. The calf is a crucial part of specialist beef production but a by-product of specialist milk production. The disjunction between producing milk and producing beef has led to calls for considering a return to dual-purpose cattle where both dairy and beef are produced from the same breed of animal, in order to reduce methane emissions. However, interviewees highlighted the infrastructural and management demands that a return to dual-purpose cattle would make.

While the impact of methane produced by sheep and beef cattle was rejected by the farmers interviewed, compliance with policy pressure, and synergies between production-oriented farming and technological measures to reduce methane meant that, at minimum, the rhetoric of methane reduction was accepted and appropriate mitigation practices adopted by some farmers.

As noted earlier, sheep and beef cattle production in the UK tends to take place in more marginal agricultural areas where environmental challenges are emphasised. It is to these that I turn next.

### Animals in environments

Despite the focus on production and animal bodies, agriculture, particularly ruminant agriculture, can rarely escape the influence of landscapes and climates (Haggerty *et al.*^2009^). As production moves to more marginal land, landscape and climate come to dominate. In stark contrast, one of the first tasks for a geneticist is to use statistical methods to separate out the genetic from the environmental, in order to understand and manipulate the genetic (with acknowledgement of possible interactions between environment and genetics). Inevitably, therefore, landscapes and climates contribute to the construction of the animal. From the farmers interviewed, three strong themes emerged, although sometimes more than one was held at the same time. The first theme emphasised the physical challenges presented by environments, the second the use of sheep and beef cattle for managing biodiversity, and the third as the complex inter-related nature of grazing animals, humans and other aspects of environments.

The restrictions placed by working in challenging environments were particularly emphasised by farmers in upland areas, where the vagaries of the weather and topography strongly influenced activities. Animals were identified as being particularly adapted to a specific environment with sheep 'hefted’ to a particular piece of land (see also Gray 1998) and managed in ways that allowed them to thrive on that land. As a result of the emphasis on the harshness of farm environment, the materiality of the animals (particularly sheep) was strongly embedded with specific locations. Farmers stressed physical aspects, such as having a robust frame to be able to cope with strong winds. Visual assessment was emphasised, as well as handling the animal in order to evaluate its condition, rather than relying on figures. There was a degree of co-operation between the farmer and the animal. Farmers asserted that sheep learn how to thrive in a specific environment from each other, but farmers assisted in animals’ adaptation to unfamiliar circumstances. For example, one farmer described how he was now teaching his sheep to eat hay, so that they would be able to rely on hay that he provided during snowy weather. During the previous snowy winter, his sheep had been unfamiliar with the hay provided for them and unwilling to eat it, even when there was no other food available.

EBVs were often viewed as appropriate for more intensive, lowland conditions without strong environmental constraints. Upland farmers emphasised how their stock was adapted to their environments, for example, although growing slowly, they would continue to grow over a longer period of time and achieve better weights than incoming animals. Incoming animals from elsewhere struggled to thrive as described by this interviewee: “*being … a very marginal hill farm…If we buy in [animals] they need a year to acclimatise anyway, so we’re far better off to keep our own [replacement animals] if we can”* (interview 116, hill sheep and beef farmer). Farmers talked about trying new breeds or new sources of their existing breeds with apparently desirable characteristics, but which proved unsuitable on their farms. For farmers in marginal areas, the challenging environments led to an emphasis on the ability of the animal to survive and remain productive in these conditions. Sheep (and to a lesser extent cattle) were considered integral to the environment, although also challenged by it.

Not only was the ability of the animals (and the farmer) to survive on hill land important for their identity, but farmer livelihood in many cases relied on payments for environmental management, particularly biodiversity conservation through various agri-environmental schemes. These schemes implied restrictions on the numbers of animals grazed, timing of grazing, and in a few cases, restrictions on providing animals with additional feed. For some, environmental management considerations were cited as reducing their freedom to change practices, e.g. *“I’m not sure what we can do to reduce it [methane], less cattle maybe but then we graze a large area and we’re driven by the number of cattle it takes to graze those marshes to a standard rather than fill in the marshes with too many cows”* (interview 95, beef farmer). These restrictions prevented farmers from considering re-seeding with less methane producing grasses, or sometimes even providing feed additives to reduce methane emissions. The animal became strongly identified with managing biodiversity.

At the extreme, biodiversity management became the main reason for keeping animals, as exemplified by one interviewee undertaking conservation grazing for an environmental body. Here again, the ability of the animals to thrive on rough pasture was emphasised, as was the ease of handling and management, for example, “*from my point of view, having animals around for longer is actually a real benefit because I observe that as they get older they become better adapted at this kind of system and most of my management problems are focused on the younger animals”* (interview 103, conservation grazer). Rapid growth is not necessary to the definition of a 'good’ animal in this context.

The emphasis on biodiversity management did not necessarily imply a co-construction of the animal and the environment. For example, an approach that emphasised efficient production could be combined with management to achieve biodiversity aims by altering the number of animals grazing rather than impacting on the animals themselves. The animal itself remains unaffected by the aspiration to manage biodiversity; rather it becomes a tool in the process. However, in other cases, such as where native breeds were selected to achieve biodiversity management, biodiversity considerations became part of the construction of the animal.

A complementary viewpoint emphasised the wider, systemic aspect of their farming approach, and the interlocked nature of the different aspects of the farming system. Animals were described as a part of complex systems which included grass, soil and trees, as exemplified by this hill farmer: “*you’re impacting one way or another on the environment in which you live, and how do you work with that, not to be against it but to work with it? … we once went down the road of having a much larger number of sheep here and I just felt, a) I was stressed, b) they were stressed c) I was overgrazing… So…we relaxed, or I did and said, wait a minute, we’ve all got to be part of this, so whether it’s a sheep or whether it’s a cow or whether it’s a deer or a hare on the hill, it seems to me the whole thing is completely interlocked*” (interview 105, hill sheep farmer).

The emphasis on farm animals as part of wider, complex systems led to an emphasis on managing methane emissions throughout these systems. Reductionist, technical approaches were viewed as inappropriate, for example “*Why breed for animals that are producing less methane when actually you could address the issues with management. In a way, producing animals that produce less methane is ignoring or avoiding the actual land management methods that can be used to control, or at least manage carbon”* (interview 112 , organic mixed farmer).

Animals were perceived as a means of providing a livelihood from a particular physical location. The physical animal was required to produce beef or sheep meat mainly or entirely from grass and to be robust to the physical conditions prevailing in the specific location. In this way the location co-produced the animal. Biodiversity could be viewed as another product that did not impact back on the animal. Rather the animal produced the biodiversity. However, animals could also be evaluated on the basis of their ability to produce biodiversity or the ease of management in circumstances where contact with humans was limited. Environmental impacts on the animal are, therefore, various, and other more complex interactions can exist, in part influenced by specific locations and farmer values.

### Animals as food

Farmers held two contrasting views on the 'good’ animal for food, depending on whether animals were sold to intermediaries or directly to consumers.

For those farmers who supplied meat direct to consumers, perceptions of consumer demand were fundamental to defining the animal. The consumer was perceived to demand natural, local and good tasting meat.

Naturalness is associated with grass fed animals. Thus, these farmers emphasised producing sheep and beef cattle from grass, in so far as was possible. Traditional, native breeds were preferred as they were perceived to be able to 'finish’ from grass (achieve an appropriate level of fat and muscle, as opposed to mere size). This preference meant that animals were bred for systems, rather than manipulating the environment to suit animals bred under different circumstances. These native breeds were perceived as able to perform well in UK conditions, unlike the 'continental’ breeds more commonly used in beef production. Less emphasis was given to growth rate, as slower growth rate was perceived as associated with better meat quality. Priority was also given to continuity of supply throughout the year (for beef), despite grass-based production systems being seasonal in their nature. Slower-growing animals provided continuing supply and were, therefore, valued as much as faster growing ones. There was no imperative to slaughter animals as quickly as possible. In constructing the animal for the direct-sales market, naturalness and continuity of supply were premium concerns.

For these farmers, the animal is co-constituted not just within its environment but in terms of how it can best meet high quality consumer desires. The cow or sheep is a link between the land and the consumer, while also remaining a part of the land, not apart from it. The technical may or may not be seen as part of this locus.

The emphasis on the natural had impacts on aspects of farm management, for example feed additives were avoided and vaccines only used when it was absolutely essential, for example, “*I’m not ill-disposed to a feed additive. What we are very careful about is just what we put in feed additives here because one of our features is that we put as little as possible into the cattle. At one stage there was also feed additives and probiotics were the feature of the month and that’s not what we want to come with our customers because when they hear feed additives they tend to go aagh, irrespective of what’s in them*” (interview 110, hill beef farmer).

An alternative view was expressed by farmers selling to intermediaries and therefore at a further distance from the consumer. These farmers were still interested in consumer perceptions, but consumer perceptions were mediated by, and often delegated to, intermediary bodies. Most farmers in this category were selling either directly to abattoirs/meat processors or selling animals at auction for purchase by meat processors. Here, the price received for the animals was a key focus of attention. Prices varied by season, demand and supply characteristics, and by weight and carcass grade. A standardised grading scheme is used for evaluating carcasses (known as the EUROP system), which is influenced by weight, visual evaluation of the fat cover on an animal and its physical shape (or conformation). The shape and fat cover on animals, therefore, become important determinants of the value of the animal to the farmer. This is reflected through to the animal breeders, who place considerable emphasis on animal shape.

In a further complication, farmers in more marginal lands are often unable to grow their animals to weights suitable for slaughter due to the unavailability of grass during the winter. Their animals are therefore sold on at lighter weights to other farmers, who grow the animals to slaughter weights. This is usually achieved through auctions of live animals during the autumn. As little information on the animals is available to purchasers, visual appraisal is predominant. Thus, the production of the 'good’ animal for slaughter has a strong emphasis on shape, weight and amount of fat cover. All these features can be visually evaluated on farm. There is therefore a strong emphasis on the materiality of the animals, but in this case emphasising its imputed 'deadstock’ value.

### Making sense of methane

Methane was perceived by the farmers interviewed as “*an inevitable consequence of keeping ruminants*” (interview 87, sheep breeder). From this perspective, as long as grass-eating, ruminating animals exist, then methane will continue to be produced. Measures to reduce methane were treated with caution with respect to the integrity of the animals as a whole, for example, “you *can’t just take methane [and] isolate it*” (interview 26, hill beef and sheep farmer). Methane was viewed as also associated with wild animals, such as deer, that would naturally inhabit the environment currently occupied by sheep and cattle. Methane was thus construed as an integral part of sheep and beef cattle and in this way, methane also becomes part of the natural environment.

Methane does not have any of the features farmers associated with a pollutant. It is not synthetically developed by humans, nor is it associated with industrial activity or combustion of hydrocarbons. When thinking about greenhouse gas emissions, farmers would refer to cars, flying, industrial pollution and in some cases intensively reared feedlot beef cattle, associated with North American production systems, for example “*the bad press was all to do with feed lot animals in California or somewhere …and that isn’t quite the same as grazing animals making use of natural vegetation”* (interview 90c, sheep breeder). Thus 'excessive’ methane is associated with perceived unnatural production methods, such as feedlot cattle systems. 'Acceptable’ methane is associated with extensive production systems that are perceived to be more 'natural’.

The image of naturalness was largely associated with the fact that sheep and (to a lesser extent) beef, unlike poultry and pigs, are predominantly fed on grass rather than cereal-based diets, for example, “*my sheep… they don’t get fed anything that makes them more gaseous, they’re just eating what’s naturally here ..So they’re just doing what they’ve always done in a natural environment*” (interview 105, hill sheep farmer). As sheep and beef cattle eat mostly grass, and the grass absorbs carbon as it grows, farmers perceived that not only are there no external inputs that would cause pollution, but that growing grass has beneficial impacts. Sheep and beef cattle farming, in their view, would be expected to reduce greenhouse gas emissions due to the carbon captured in grass, or at minimum to be carbon neutral, rather than a source of greenhouse gases. Thus, arguments about the large climate change impact of sheep and beef cattle were not felt to be credible.

Extensive production systems were associated with traditional cultural practices and hence represent a human-animal association that has existed over historical periods of time. Since methane from these production systems has not caused a problem in the past, there was no expectation that it will cause problems in the future. Reference was made to the 'wisdom’ of nature that has ensured that traditional sheep and beef cattle farming is in harmony with other factors in nature, as exemplified in this quote “*The whole world has been covered in ruminants from time began… Mother Nature, whatever that is, wouldn’t have created a system that was going to destroy itself*” (interview 98, beef breeder). Rather than being a modern invention, sheep and beef cattle are part of an historic, natural order.

In the rhetoric of the farmers interviewed, sheep and beef cattle are not only a natural feature of the landscape but also have an important role in producing food for humans, managing the environment and providing a source of income, particularly in hill regions where there are perceived to be few other options for making a living, as exemplified in the following quote:

“*You want to go up to where [my wife] comes from and see if you can grow crops up there, good luck … Steepness of slope, soil type, height above sea level, all those sorts of things just means there are vast areas that you can’t grow crops on, so we have to utilise the grass that we can grow*” (interview 30, dairy and sheep farmer).

These arguments enlist sheep and beef cattle farming into the sphere of the moral 'good’. Farmers drew on repertoires of the intrinsic positive character of their management of nature in contrast to unnatural practices, as evidenced in the following quote:

*“I can’t see what’s wrong with what we’re doing, we’re producing a product that we can eat from a hill that otherwise would be either forestry, originally it would be forestry… I think we’re doing a natural rearing method and I don’t personally feel wrong about doing it, I feel confident that it’s a good and right thing to do… I think there’s plenty of other things in modern trappings that are doing more harm than that”* (interview 117, hill sheep farmer).

The evidence from these interviews suggests that there is little acceptance of methane emissions from sheep and cattle as a 'problem’ or 'pollutant’ requiring action by farmers. This message was remarkably consistent across all the production systems studied, including farmers with an inherent ideology of environmental care, such as some organic farmers. Methane was understood as an integral part of sheep and beef cattle and a necessary part of their grass-eating identity. Moreover, methane was seen as part of the specific farming context, particularly in the uplands, resonating with the culture of 'natural’ production and the historical and cultural practices associated with these farming systems. Thus, to subvert Mary Douglas’s (1966) famous description of pollution as 'matter out of place’, for sheep and beef cattle farmers in the UK, methane is very much 'matter in place’. Sheep and beef cattle were seen as having a natural role in relation to humans, and methane as an inevitable by-product of this role. In the words of one hill sheep farmer, sheep (and by inference cattle) should be “*allowed their place in society”* (interview 21, sheep and beef farmer). In defining the grazing animal, methane production is also defined.

Methane was primarily conceptualised as an inevitable and natural part of sheep and beef cattle. As these animals contribute to the good of humankind by converting grass to a product edible by humans, there is a normative justification to allowing sheep and beef cattle to continue to exist. From a social perspective, farmers felt methane emissions were yet another criticism to be heaped onto beleaguered farmers who had more pressing issues to deal with. Arguments for the climate change impact of methane based on codified knowledge and scientific best estimates was perceived to be in conflict with knowledge that views methane emissions in the context of carbon cycles that take into account absorption by grass and the lack of introduction of 'artificial’ factors.

### Contextualising co-productions

It is perhaps trite, but nevertheless important, to note that not all farmers or farms are the same. This is salient as the environmental context, market and ethos of the farm provide the location within which both the animal and the technical are constituted, and which therefore impact upon the way constructions are made.

Co-productions do not take place in isolated, sterile circumstances but are redolent with history of interactions among animals, farmers and technical specialists (Reardon 2001). In the case of the farmers interviewed here, a strong sense of historic injustice was communicated. Farmers felt they had adopted measures on advice from technical specialists, but these measured turned out to be disadvantageous, for example, “*Agriculture has always been pretty quick to take on board new things that were deemed to be good, but we’ve had quite a lot of stuff come at us that’s deemed to bad, well it’s turned out to be bad”* (interview 26, hill beef and sheep farmer). Farmers articulated examples from their experiences of BSE, Foot and Mouth Disease and environmental management - measures they had been asked to put in place and were now being asked to remove. They mentioned perceived bad genetics advice given to the dairy industry, resulting in lame and infertile cows due to excessive emphasis on milk yield. These experiences led to farmers’ scepticism regarding technical expertise and reluctance to appropriate such knowledge in their practices.

The current framing of methane from farm animals assumes that methane is primarily the producers’ rather than the consumers’ concern. In purchasing white goods or cars, an energy rating is provided on appliances or efficiency figures on cars, providing the consumer with information that can enable choice. Yet in purchasing meat, choices are largely restricted to choosing the species (e.g. buying lamb or chicken) or responding to exhortations to reduce meat consumption. There seems to be little link between 'reduced methane’ and consumers of sheep meat and beef. The benefits of reducing methane are expected to be gained purely from improved production and hence expected improved profitability for farmers. If the evidence of Barr and colleagues holds (Barr *et al.*^2011^), consumers are taking little responsibility for climate change through their meat purchases and, therefore, are unlikely to demand low carbon footprint meat. However, consumer purchasing responses could readily change under different conditions such as pricing, availability, information, social pressures and so on. None of which are explored here.

The focus in the policy world on measuring quantity of greenhouse gas emissions means that the method of accounting for greenhouse gas emissions becomes crucial to understanding where policy levers can best be applied. Current practice is to use relatively crude calculations that impute a standard amount of methane emissions to each animal. Effectively it is the number of ruminant animals that are being measured, rather than methane emissions *per se*. For example, reductions of greenhouse gas emissions of 21% between 1990 and 2008 (Committee on Climate Change 2010) are explained as primarily due to a drop in the total number of animals, largely due to changes in the support regime that replaced payments for each animal with a Single Farm Payment (Gill *et al.*^2010^). So, while the rhetoric of the policy world is to reduce methane emissions, the practice is to reduce the number of animals. At this point, there is a synergy between farmer and policy concepts, each understanding animals as a methane producing entity. While farmers struggle to view methane as being amenable to technological manipulation, policy makers look to more discriminating measures of methane that could take the impacts of technological manipulation into account.

From a scientific perspective, reflected in the views of non-farming interviewees, methane emissions are readily manipulable. Methods have been identified that have the potential to reduce emissions, with the added benefit of increased efficiency of output. In contrast, as described above, sheep and beef cattle are located in specific natural environments, production systems and markets and subject to appropriate practices and norms. Introduction of methane reduction as a new norm requires the introduction of a new form of social order that recognises low methane production as part of the constitution of a 'good’ animal. In productivist approaches, this is already achieved through attention paid to efficiency of production, but in other approaches, particularly those which give high value to concepts of the 'natural’, the association of methane reduction with intensification is anathema. The implication on evaluating the 'good’ animal is that methane will continue to be constructed as a natural and inevitable part of the animal which is not a target for technological manipulation. If methane reductions are to be realised, conceptually this can only be achieved through reducing the number of sheep and beef cattle. At this point the farming and policy worlds again merge, but in ways that are threatening to the livelihoods of farmers.

## Conclusions

This paper extends the concept of co-production as an analytical framework for understanding the evaluation of a 'good’ animal to encompass methane. As noted earlier, the sheep and beef farming sector tends not to be strongly influenced by technology (although exceptional farms exist).The construction of an animal is predicated on a range of other factors, in particular the environment and consumer market orientation. However, across a diverse range of farming approaches, it became very apparent that farmers have difficulty in accepting the role of sheep and beef cattle in climate change. Methane emissions were viewed as normal and natural.

The policy imperative to reduce greenhouse gas emissions has resulted in the identification of a number of technical approaches to reducing methane, requiring action by farmers. These technological approaches presume that the animal can be separated from its methane emissions. However, from farmers’ perspectives, methane emissions are an integral part of the animal and, therefore, difficult to conceptualise as amenable to technological manipulation.

Unlike most pigs and chickens which tend to be kept in uniform environmental conditions, sheep and beef cattle farming is characterised by heavy reliance on grazing grass, often in challenging climatic and topographical situations. The environment, therefore, becomes an important part of the construction of sheep and beef cattle. Animals are literally selected for survival by their environments. This local and specific conceptualisation of animals contrasts with the presumption that technologies can be applied across all environments. Technologies that reduce methane emissions by increasing productivity are synergistic with farming systems where efficiency of production is emphasised only as long as other values and aspirations are not compromised. Methane reduction has tended to become associated with paradigms of industrialised agriculture emphasising the utilitarian functions of animals and this is anathema to farmers aiming at more holistic approaches.

The range of different markets to which the meat animal is sold also has a strong impact on the definition of the 'good’ animal; with emphasis on naturalness and links to the local for sales direct to consumers, and emphasis on shape and fatness for sales to slaughterhouses. Consumer responsibilities have largely been limited to exhortations to reduce consumption of red meat. The moral responsibility has thus largely been shifted from consumers to producers, with little evidence of demand from consumers to apply technology to animals to reduce methane emissions.

The sheep and beef cattle context involves complex assemblages of animals, nature, markets and technologies to providing a portfolio of different social and private 'goods’ depending on the farmers own values and circumstances. This paper identifies exemplars of how contextual factors dramatically impact on animal co-productions highlighting a serious mismatch between policy/scientific constructions and farmer constructions. I suggest that to include methane in the category 'manipulable by technology’ requires more sophisticated co-construction of the animal and technology. While application of technology is one possible policy approach to reducing methane, alternative methods, such as reducing demand for meat, or balancing stocking rates with carbon absorption capacity, need not be neglected.

## Endnotes

^a^Numbering is retained from automatic numbering on the recording device in order to maintain traceability of evidence. Hence the numbers allocated do not start at 0 and therefore exceed 42.

## Author’s information

AB is a Research Fellow at the University of Edinburgh holding post-graduate degrees in both innovation studies and animal genetics. As well as lecturing, she is currently employed as an Agri-Food Knowledge-Exchange Fellow with the Natural Environment Research Council.

## References

[CR1] Barnes AP, Toma L (2012). A typology of dairy farmer perceptions towards climate change. Climate Change.

[CR2] Barr S, Shaw G, Coles T (2011). Sustainable lifestyles: sites, practices and policy. Environ Plann A.

[CR3] Blackstock KL, Ingram J, Burton R, Brown KM, Slee B (2010). Understanding and influencing behaviour change by farmers to improve water quality. Sci Total Environ.

[CR4] Burton RJF, Kuvzera C, Schwarz G (2008). Exploring Farmer’s cultural resistance to voluntary agri-environmental schemes. Sociol Rural.

[CR5] Calvert S (2013). Certified angus, certified patriot: breeding, bodies and pedigree practices. Sci Culture.

[CR6] Committee on Climate Change (2010). The Fourth Carbon Budget. Reducing emissions through the 2020s.

[CR7] Convery IT, Bailey C, Mort M, Baxter J (2004). Death in the wrong place? emotional geographies of the UK 2001 foot and mouth disease epidemic. J Rural Stud.

[CR8] Doubleday R (2007). Organising accountability: co-productions of technoscientific and social worlds in a nanoscience laboratory. Area.

[CR9] Douglas M (1966). Purity and Danger: An analysis of the Concepts of Pollution and Taboo.

[CR10] Eden S, Bear C, Walker G (2008). Mucky carrots and other proxies: problematising the knowledge-fix for sustainable and ethical consumption. Geoforum.

[CR11] Evans N, Gaskell P, Winter M (2003). Re-assessing agrarian policy and practice in local environmental management: the case of beef cattle. Land Use Policy.

[CR12] Foresight. The Future of Food and Farming (2011). Final Project Report.

[CR13] Garnett T (2011). Where are the best opportunities for reducing greenhouse gas emissions in the food system (including the food chain)?. Food Policy.

[CR14] Garnett T, Godfray C (2012). Sustainable intensification in agriculture. Navigating a course through competing food system priorities.

[CR15] Genesis Faraday Partnership (2008). A study of the scope for the application of research in animal genomics and breeding to reduce nitrogen and methane emissions from livestock based food chains.

[CR16] Gibbs D, Holloway L, Gilna B, Morris C (2009). Genetic techniques for livestock breeding: restructuring institutional relationships in agriculture. Geoforum.

[CR17] Gilg A, Barr S, Ford N (2005). Green consumption or sustainable lifestyles? Identifying the sustainable consumer. Futures.

[CR18] Gill M, Smith P, Wilkinson JM (2010). Mitigating climate change: the role of domestic livestock. Animal.

[CR19] Goodman D (1999). Agro-food studies in the 'Age of Ecology’: nature, corporeality, Bio-politics. Sociol Rural.

[CR20] Grasseni C (2007). Managing cows: an ethnography of breeding practices and uses of reproductive technology in contemporary dairy farming in Lombardy (Italy). Stud Hist Philos Biol Biomed Sci.

[CR21] Gray J (1998). Family farms in the Scottish borders: a practical definition by hill sheep farmers. J Rural Stud.

[CR22] Green K, Foster C (2005). Give peas a chance: transformations in food consumption and production systems. Technol Forecasting Soc Change.

[CR23] Haden VR, Niles MT, Lubell M, Perlman J, Jackson LE (2012). Global and Local Concerns: What Attitudes and Beliefs Motivate Farmers to Mitigate and Adapt to Climate Change?.

[CR24] Haggerty J, Campbell H, Morris C (2009). Keeping the stress off the sheep? Agricultural intensification neoliberalism, and 'good’ farming in New Zealand. Geoforum.

[CR25] Holloway L (1999). Understanding climate change and farming: scientific and farmers’ constructions of 'global warming’ in relation to agriculture. Environ Plann A.

[CR26] Holloway L (2004). Aesthetics, genetics, and evaluating animal bodies: locating and displacing cattle on show and in figures. Environ Plann: Soc Space.

[CR27] Holloway L (2007). Subjecting cows to robots: farming technologies and the making of animal subjects. Environ Plann D: Soc Space.

[CR28] Holloway L, Bear C (2011). Commentary. Environ Plann A.

[CR29] Holloway LE, Ilbery BW (1996). Famers’ attitudes towards environmental change, particularly global warming, and the adjustment of crop mix and farm management. Appl Geogr.

[CR30] Holloway L, Morris C (2008). Boosted bodies: genetic techniques, domestic livestock bodies and complex representations of life. Geoforum.

[CR31] Holloway L, Morris C, Gilna B, Gibbs D (2009). Biopower, genetics and livestock breeding: (re)constituting animal populations and heterogeneous biosocial collectivities. Trans Ins British Geo NS.

[CR32] Holloway L, Morris C, Gilna B, Gibbs D (2011). Choosing and rejecting cattle and sheep: changing discourses of practices of (de)selection in pedigree livestock breeding. Agric Hum Values.

[CR33] Holstein JA, Gubrium JF, Weinberg D (2002). Active Interviewing. Qualitative Research Methods.

[CR34] Industry Delivery Partners (2011). Meeting the Challenge: Agriculture Industry GHG Action Plan Phase 1: 2010–2012 4th April.

[CR35] Jasanoff S, Jasanoff S (2006). The idiom of co-production. States of Knowledge. The co-production of science and social order.

[CR36] Jones AK, Jones DL, Edward-Jones G, Cross P (2013). Informing decision making in agricultural greenhouse gas mitigation policy: a best-worst scaling survey of expert and farmer opinion in the sheep industry. Environ Sci Pol.

[CR37] Kahneman D, Tversky A (1982). Judgement under Uncertainty.

[CR38] Lowe P, Cark J, Seymour S, Wars N (1997). Moralizing the Environment: Countryside Change, Farming and Pollution.

[CR39] Macnaghten P (2004). Animals in their nature: a case study on public attitudes to animals, genetic modification and 'Nature’. Sociology.

[CR40] Moran D, Macleod M, Wall E, Ivery V, McVittie A, Barnes A, Rees R, Topp CFE, Moxey A (2011). Marginal abatement cost curves for UK agricultural greenhouse Gas emissions. J Agric Econ.

[CR41] Murdoch J, Marsden T, Banks J (2000). Quality, nature and embeddedness: some theoretical considerations in the context of the food sector. Econ Geogr.

[CR42] Reardon J (2001). The human genome diversity project: a case study in coproduction. Soc Stud Sci.

[CR43] Riley M (2011). 'Letting them go’ – agricultural retirement and human-livestock relations. Geoforum.

[CR44] Ritchie J, Lewis J, Elam G, Ritchie J, Lewis J (2003). Designing and Selecting Samples. Qualitative Research Practice. A Guide for Social Science Students and Researchers.

[CR45] Saltzman K, Head L, Stenseke M (2011). Do cows belong in nature? The cultural basis of agriculture in Sweden and Australia. J Rural Stud.

[CR46] Schot J, Rip A (1996). The past and future of constructive technology assessment. Technol Forecast Soc Chang.

[CR47] Spence A, Pidgeon N (2010). Framing and communicating climate change: the effects of distance and outcome frame manipulations. Glob Environ Chang.

[CR48] Spence A, Poortinga W, Pidgeon N, Lorenzoni I (2010). Public perceptions of energy choices: the influence of beliefs about climate change and the environment. Energy Environ.

[CR49] Stassart PM, Whatmore SJ (2003). Metabolising risk: food scares and the un/re-making of Belgian beef. Environ Plann A.

[CR50] Steinfeld H, Gerber P, Wassenaar T, Castel V, Rosales M, de Haan C (2006). Livestock’s Long Shadow: environmental issues and options.

[CR51] Ward N, Lowe P, Seymour S, Clark J (1995). Rural restructuring and the regulation of farm pollution. Environ Plann A.

[CR52] Ward N, Clark J, Lowe P, Seymour S (1998). Keeping matter in its place: pollution regulation and the reconfiguring of farmers and farming. Environ Plan.

[CR53] Wilkie R (2005). Sentient commodities and productive paradoxes: the ambiguous nature of human-livestock relations in Northeast Scotland. J Rural Stud.

[CR54] Yarwood R, Evans N (2003). Livestock, locality and landscape: EU regulations and the new geography of Welsh farm animals. Appl Geogr.

[CR55] Yarwood R, Evans N (2006). A Lleyn sweep for local sheep? Breed societies and the geographies of Welsh livestock. Environ Plann A.

